# Single crystal growth, optical absorption and luminescence properties under VUV-UV synchrotron excitation of type III Ce^3+^:KGd(PO_3_)_4_, a promising scintillator material

**DOI:** 10.1038/s41598-018-29372-z

**Published:** 2018-07-20

**Authors:** Irina Adell, Rosa Maria Solé, Maria Cinta Pujol, Matthieu Lancry, Nadège Ollier, Magdalena Aguiló, Francesc Díaz

**Affiliations:** 1 Universitat Rovira i Virgili, Departament Química Física i Inorgànica, Física i Cristal·lografia de Materials i Nanomaterials (FiCMA-FiCNA) - EMaS, Campus Sescelades, c/Marcel·lí Domingo, 1, E-43007 Tarragona, Spain; 2 Institut de Chimie Moléculaire et des Matériaux d’Orsay, CNRS-Université Paris Sud, Université de Paris Saclay, Bât.410, 91405 Orsay, France; 3 Laboratoire des Solides Irradiés, CEA-CNRS-Ecole Polytechnique, Université Paris-Saclay, Palaiseau, France

## Abstract

Scintillator materials have gained great interest for many applications, among which the medical applications stand out. Nowadays, the research is focused on finding new scintillator materials with properties that suit the needs of each application. In particular, for medical diagnosis a fast and intense response under high-energy radiation excitation is of great importance. Here, type III Ce^3+^-doped KGd(PO_3_)_4_ single crystals with high crystalline quality are grown and optically characterized as a new promising scintillator material. The 4*f* → 5*d* electronic transitions of Ce^3+^ are identified by optical absorption. The optical absorption cross section of Ce^3+^ for the electronic transition from the ^2^F_5/2_ to the 5*d*_1_ level is 370 × 10^−20^ cm^2^. The luminescence of KGd_0.996_Ce_0.004_(PO_3_)_4_ crystal by exciting the 5*d* levels of Ce^3+^ with VUV-UV synchrotron radiation shows down-shifting properties with strong emissions at 322 and 342 nm from the 5*d*_1_ to ^2^F_5/2_ and ^2^F_7/2_ levels of Ce^3+^ with a short decay time of ~16 ns, which is very suitable for scintillator applications. Moreover, these intense emissions are also observed when Gd^3+^ is excited since an energy transfer from Gd^3+^ to Ce^3+^ exists.

## Introduction

Scintillator materials have gained great interest for a large number of applications, such as in medical imaging techniques (X-ray computed tomography, positron emission tomography), high energy and nuclear physics, non-destructive testing, amongst others^1–4^. An ideal scintillator material should satisfy different properties, mainly high density, fast emission and a high light yield^5^. Many efforts are dedicated to find novel scintillator materials with the best properties for different applications, especially in the case of medical applications with the aim to reduce the ionizing radiation exposure to the patient in diagnostic techniques^6^.

In scintillator materials which contain lanthanide ions, the emissions in the ultraviolet and visible regions suitable for scintillation applications are produced mainly by the 5*d* → 4*f* electronic transitions of these ions, which act as scintillator centres. Typical scintillator materials are Ce^3+^-doped crystals, such as Ce^3+^:Lu_2_SiO_5_ and Ce^3+^:LaBr_3_. Both crystals present some drawbacks, such as difficulty for crystal growth and hygroscopicity, respectively^7,8^. Multicomponent garnet crystals doped with Ce^3+^ are also used in medical imaging and gamma spectroscopy due to high light yield and high density, but with poorer timing performance compared with lutetium-yttrium oxyorthosilicate crystals^9^. Ce^3+^ emission bands are based on the 5*d* to 4*f* electronic level transitions, usually as a doublet since the arrival states, ^2^F_5/2_ and ^2^F_7/2_, are separated by around 2000 cm^−1^ ^10^. These electronic transitions are allowed and have a very short decay time in the order of nanoseconds^11^, which could be suitable for scintillator applications.

Crystalline alkali and rare earth polyphosphates, MRE(PO_3_)_4_ (where M is an alkali cation, M = Li, Na and K; RE is a *r*are *e*arth cation), have been recently studied as candidates for scintillator applications^2^ due to their broad transparency range in the ultraviolet region, its relatively high density and the possibility to incorporate Ce^3+^ and Pr^3+^ ions which are characterized by fast emissions in many hosts. They have been also intensively studied for other optical applications due to the low concentration quenching of the luminescence at high concentration of the active Ln^3+^ ions^12^. Ce^3+^ luminescence in phosphate compounds have been deeply studied, by Blasse and Dirken^13^ in LiLaP_4_O_12_, by Shalapska *et al*.^12^ in LiYP_4_O_12_, Zhong *et al*.^14^ in 2007 in AGdP_4_O_12_ (being A = Cs, K, Na and Li) and Novais *et al*.^15^ in 2014 in the non-centrosymmetric monoclinic phase NaYP_2_O_7_.

Among them, KGd(PO_3_)_4_ is a suitable crystal to incorporate different Ln^3+^ ions substituting the Gd^3+^ ions of the structure, is not hygroscopic, presents a good chemical stability^16^ and its density is 3.538 mg·m^−3^ ^17^. Besides, it is a deep-ultraviolet crystal closing at 160 nm^18^ indicating a large band gap, which potentially could allow that the 5*d* energy levels of some Ln^3+^ ions be located between the conduction band and the valence band.

The existence of the radioisotope ^40^K (0.0117% natural abundance) may limit its usefulness for some applications by increasing the background radiation. However, as mentioned in the review of Nikl^19^, other radioactive isotopes are also present in several used scintillator crystals such as ^176^Lu, ^87^Rb, and ^138^La contributing in a background signal. Besides, other promising scintillator materials identified in the literature also contain K as ion, such as: K_2_LaI_5_:Ce^3+^, being attractive by its fast decay time of 24 ns, together with a relative high density^20^; and KSr_2_I_5_:Eu^2+^ with a very high yield^21^.

Finally, we have already reported the feasibility of growing these crystals within the non-centrosymmetric monoclinic crystalline phase with high crystalline quality^18,22^. This compound presents a polymorphism related to the degree of condensation of the phosphoric anions: the PO_4_ forms chains in the type III phase (space group: *P*2_1_) and type IV phase (space group: *P*2_1_/*n)*, belonging to the group of polyphosphates, and the PO_4_ forms rings in the type B phase (space group: *C*2/*c*) belonging to the group of cyclophosphates^22^. High quality bulk single crystals of Ln^3+^-doped non-centrosymmetric KGd(PO_3_)_4_ (space group: *P2*_1_) have been already grown by us by the Top Seeded Solution Growth technique. The unit cell parameters for undoped KGd(PO_3_)_4_ are *a* = 7.255(4) Å, *b* = 8.356(5) Å, *c* = 7.934(5) Å, β = 91.68(5)° and Z = 2 refined by single crystal X-ray diffraction^17^.

Potassium and Ce^3+^ stoichiometric phosphate, KCe(PO_3_)_4_, also presents polymorphism; in this compound, the phosphoric anions can form chains in the type III phase (space group: *P*2_1_)^23^. Since KCe(PO_3_)_4_ also presents the type III crystalline phase, it is expected that the non-centrosymmetric phase (type III) KGd(PO_3_)_4_ can be doped with high concentration of Ce^3+^.

Thus, KGd(PO_3_)_4_ as a host and Ce^3+^ as doping element (hereafter Ce^3+^:KGdP) is a promising combination to obtain a new scintillator material. The aim of our research is to growth non-centrosymmetric Ce:KGdP single crystals from high temperature solutions with different Ce^3+^ doping concentrations in order to carry out a study of the luminescence properties of these crystals under synchrotron VUV excitation and discuss the potential of this crystalline phase of KGdP for scintillator applications.

## Results and Discussion

### Bulk single crystal growth

Table 1 summarizes the growth conditions of the different single crystal growth experiments carried out in this work and some features of the crystals obtained.

**Table 1:** Crystal growth conditions and crystals obtained from about 100 g of solution.

Exp. number	[Ce_2_O_3_]/([Gd_2_O_3_] + [Ce_2_O_3_]) in the solution [at.%]	Growth interval [K]	Crystal weight [g]	Crystal dimensions along a*×*b* × c* directions [mm]	Growth rate [ × 10^−3^ g·h^−1^]
1	0.25	27.4	0.62	3.1 × 9.9 × 9.9	1.55
2	0.50	30	1.62	6.1 × 15.1 × 10.9	3.61
3	0.50	29	1.65	5.4 × 14.9 × 11.4	3.84
4	1.00	31	1.31	3.9 × 13.2 × 12.2	2.78
5	1.00	32	1.76	6.0 × 15.7 × 11.5	3.58
6	2.00	34.3	1.68	4.9 × 15.5 × 13.3	3.25

The saturation temperature of the solutions was determined to be at around 950 K. As expected this saturation temperature is similar to the one reported for the crystal growth of Yb-doped KGdP crystals, grown in similar experimental conditions^24^ and lower than reported for undoped KGdP^16^. Lower crystallization temperature would mean higher viscosity in the solution, which can affect negatively to the crystal quality and the growth rate of the crystals.

These crystals were colourless, transparent, and generally free from inclusions and cracks. The size of the crystals obtained along a* × *b* × c* directions were 3.1–6.1 × 9.9–15.7 × 9.9–13.3 mm and the weight of the crystals ranged from 0.62 to 1.76 g. In all cases, the crystal dimension along the *b* crystallographic axis was higher than the dimensions in a* and c* directions. Comparing the a* dimension with previous reported grown crystals, in this case the dimensions are smaller. One of the reasons of these smaller crystal dimensions is the lower amount of growth solution in the crucible due to the smaller crucible diameter. In addition, changes in the thermal vertical gradient, especially in the first centimetre of the solution, and different position of the stirrer in relation to the crystal growing can also contribute to produce changes in the crystal dimensions.

The crystal growth rates were generally higher than 3 × 10^−3^ g·h^−1^. In previous reports, the crystal growth rate was around 8-9 × 10^−3^ g·h^−1^ for undoped KGdP^25^ and around 7 × 10^−3^ g·h^−1^ for Nd-doped KGdP^18^. Several reasons can induce this slower crystal growth, which are the different composition of the solution with a reduced amount of solute in it, the total mass of the solution which in the present work is lower, and finally, the presence of cerium in the solution. However, it is significant to point out the difficulty of the growing high quality crystals due to the high viscosity of the solution, around 19 Pa·s at 950 K, and how by choosing and designing accurate crystal growth conditions, such as appropriate stirring of the solution by the use of stirrer and cooling ramps, high crystalline quality crystals have been obtained. Crystal growth is very difficult in highly viscous solutions, since due to the low molecular mobility inside them, the growth units find difficulties to reach the crystal surface.

**Figure 1:**
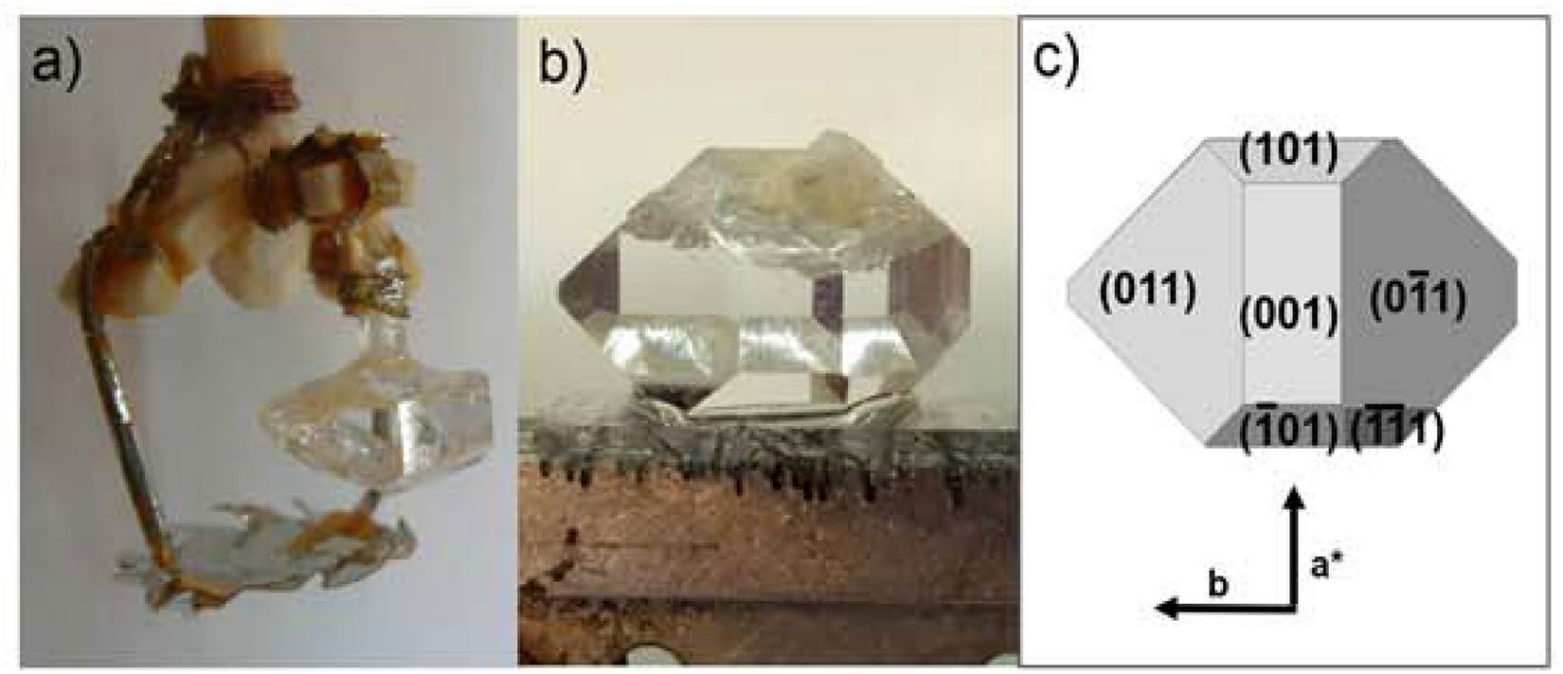
(**a**) As-grown single crystal of Ce^3+^:KGdP and platinum stirrer, (**b**) As-grown single crystal of Ce^3+^:KGdP and (**c**) Crystal scheme with the faces observed.

As an example, Fig. 1 shows an as-grown Ce^3+^:KGdP single crystal with the platinum stirrer and a morphological scheme with the crystalline faces observed in this crystal. The crystal scheme corresponds to the crystalline habit of type III phase of KGdP.

The distribution coefficient of Ce^3+^ in KGdP was calculated using the EPMA results according to the formula: K_Ce_ = ([Ce]/([Ce] + [Gd]))_crystal_/([Ce]/([Ce] + [Gd]))_solution_. The stoichiometry of the crystals and the Ce^3+^ distribution coefficients are shown in Table 2. The cerium distribution coefficient is larger than one; this means that the Ce^3+^ distribution inside the crystal may be not homogeneous. Besides, the distribution coefficient of Ce^3+^ is larger than the observed one for Yb and similar to the one reported for Nd^18,24^. This last fact can be related to the different ionic radii of the lanthanide doping ions.

**Table 2:** EPMA results for Ce:KGdP. *K*_Ce_ denotes the distribution coefficient of the Ce^3+^ in the crystal.

Ce^3+^ at.% in solution	*K*_Ce_	Ce^3+^ concentration [cm^−3^]	Chemical formula
0.25	1.77	1.667 × 10^19^	KGd_0.996_Ce_0.004_(PO_3_)_4_
0.5	1.30	2.501 × 10^19^	KGd_0.994_Ce_0.006_(PO_3_)_4_
1	1.88	7.919 × 10^19^	KGd_0.981_Ce_0.019_(PO_3_)_4_
2	1.28	1.084 × 10^20^	KGd_0.974_Ce_0.026_(PO_3_)_4_

In addition, X-ray powder diffraction analysis was made to study the effect on the unit cell parameters of KGdP when it is doped with Ce^3+^ ions. For undoped KGdP, with non-centrosymmetric crystalline structure, the unit cell parameters are *a* = 7.2510(4) Å, *b* = 8.3498(2) Å, *c* = 7.9240(2) Å, β = 91.823(3)°, with Z = 2 and the unit cell volume is 479.51(3) Å^3^. In the case of KCe_0.026_Gd_0.974_(PO_3_)_4_ crystals, the unit cell parameters are *a* = 7.2520(4) Å, *b* = 8.3524(2) Å, *c* = 7.9265(2) Å, β = 91.826(3)°, with Z = 2 and the unit cell volume is 479.88(3) Å^3^. As can be seen, the unit cell parameters and the unit cell volume slightly increase when KGdP is doped with Ce^3+^ ions. This is expected since the ionic radius of Ce^3+^ with coordination VIII is higher than the ionic radius of Gd^3+^ with the same coordination (1.143 Å and 1.053 Å, respectively)^26^. Moreover, by acquiring the X-ray diffraction pattern for obtaining the unit cell parameters of the Ce^3+^-doped crystals, it is proved that the crystalline phase of the crystals is the type III one (space group: *P*2_1_).

### Ce^3+^ spectroscopy in KGd(PO_3_)_4_ single crystals

#### Optical absorption

As it is known, the 5*d* electrons present a strong interaction with the crystal field, which determines the position of the 5*d* energy levels and its high dependence with the crystal host^27–29^. These bands are also wide due to the 5*d* electron interaction with the lattice phonons. In the non-centrosymmetric KGdP crystalline phase, Ce^3+^ substitutes the Gd^3+^ ions in a C_1_ position inside a GdO_8_ distorted dodecahedra. The 5*d* level splits in five non-degenerated crystal-field levels.

**Figure 2:**
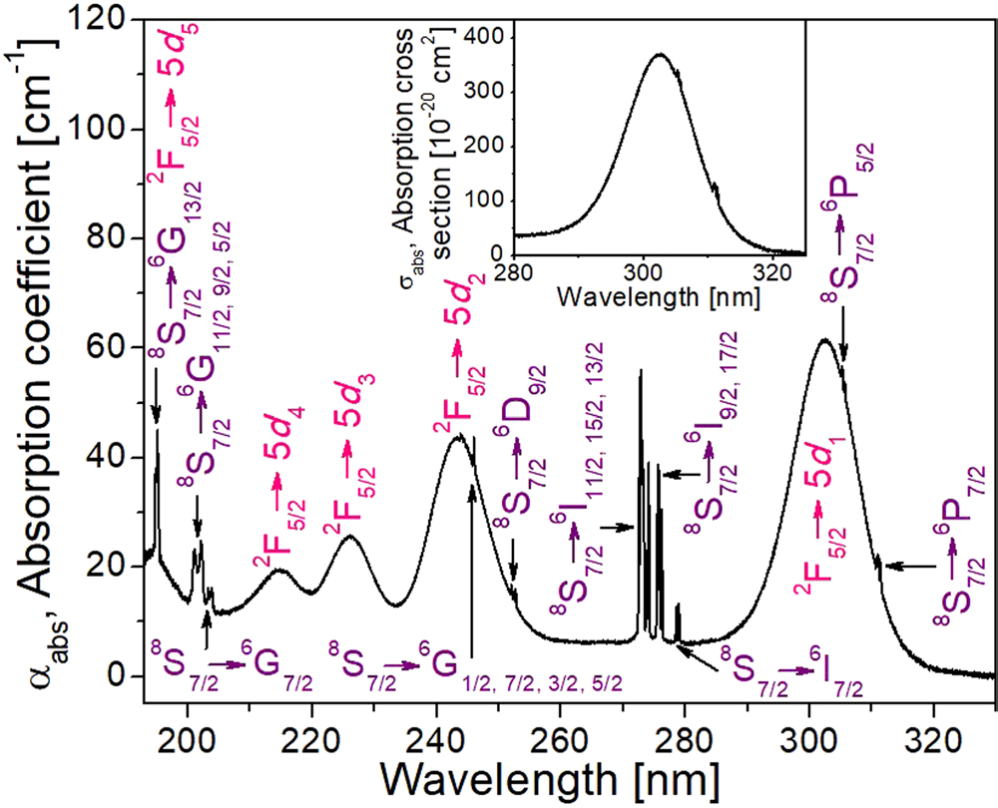
Unpolarised optical absorption coefficient of KGd_0.996_Ce_0.004_(PO_3_)_4_ at room temperature. Propagation direction is along *b* axis. Labels in purple indicate the absorption transitions of Gd^3+^ and labels in pink those of Ce^3+^. Inset: Unpolarised optical absorption cross section of ^2^F_5/2_→5*d*_1_ transition of Ce^3+^ in KGd_0.996_Ce_0.004_(PO_3_)_4_ at room temperature.

Figure 2 shows the unpolarised optical absorption coefficient of KGd_0.996_Ce_0.004_(PO_3_)_4_ at room temperature and the inset displays the unpolarised optical absorption cross section of ^2^F_5/2_→5*d*_1_ transition of Ce^3+^ in the same crystal at room temperature. As can be seen in this Figure, the strongest bands are assigned to the 4*f*→5*d* vibronic transitions. The wavelengths assigned to each 4*f*→5*d*_1_, 5*d*_2_, 5*d*_3_, 5*d*_4_ and 5*d*_5_ absorptions are resumed in Table 3. The wavelength values for the 4*f*→5*d* electronic transitions in other hosts are also summarised in Table 3.

**Table 3:** Spectroscopic properties and crystallographic space groups of Ce^3+^ doped phosphates.

Compound	Space group	R_av_ (Gd-O) [Å]	CF	λ_5_, λ_4_, λ_3_, λ_2_, λ_1_ [nm]	*D*(A)[cm^−1^]	ε_cfs_ [cm^−1^]	ε_c_[cm^−1^]	R_eff_ [Å]	α_sp_ [10^−30^ m^3^]	Ref.
KGd_0.996_Ce_0.004_(PO_3_)_4_	*P*2_1_	2.4065	ddh	194, 215, 226, 243.5, 302.5	16282	18489	7944	2.447	1.479	This work
KGd_0.99_Ce_0.01_P_4_O_12_	*C*2/*c*	2.408	ddh	193, 209, 221, 245, 307	16767	19240	7570	2.453	1.431	^14^
NaGd_0.99_Ce_0.01_(PO_3_)_4_	*P*2_1_/*n*	— 2.401	— ddh	—, —, 223, 256, 297 196, 207, 218, 254, 297	— 15670	— 17350	— 7582	— 2.443	— 1.398	^2, 14^
NaPr_0.9_Ce_0.1_P_4_O_12_	*P*2_1_/*n*	—	ddh	195, 230, 252, 268, 295	15442	17300	7332	—	—	^12^
LiLa_0.95_Ce_0.05_P_4_O_12_	*C*2/*c*	—	ddh	—, —, —, —, 278	13369	—	—	—	—	^13^

Note: R_av_ = average distance, CF = coordination figure, ddh = dodecahedron, λ_5_, λ_4_, λ_3_, λ_2_, λ_1_ = absorption bands of the 5*d* levels, *D*(A) = spectroscopic redshift, ε_c_ = centroid shift_,_ ε_cfs_ = crystal field splitting, R_eff_ = effective distance of Gd-O, and α_sp_ = spectroscopic polarizability.

The broad band centred at 302.5 nm (33058 cm^−1^, 4.10 eV) (see Fig. 2) corresponds to the first spin-allowed 4*f*→5*d* transition from ground state to the first 5*d* level (5*d*_1_) of Ce^3+^ in the KGdP host. This value is very close to the reported values in other crystalline phosphates, such as 310 nm for K_3_La(PO_4_)_2_^30^, and 307 nm for the centrosymmetric KGdP (space group: *C*2/*c*)^14^. It is worth to point out that the photons at 194, 244 and 302.5 nm excite both Ce^3+^ and Gd^3+^ ions; whereas the other identified wavelengths, 214 and 226 nm, excite predominantly only Ce^3+^ ions.

The centroid position of the 5*d* energy levels of Ce^3+^ in a free ion is 51230 cm^−1^ ^28^. Due to the large interaction of the 5*d* electron with the crystal field of the host A, the first dipole allowed 4*f*→5*d* transition is shifted in relation with the free ion value; this shift is defined as the spectroscopic redshift, *D*(A)^28^. The spectroscopic redshift of Ce^3+^ due to the type III KGdP host is *D*(KGdP) = 16282 cm^−1^. As mentioned by Dorenbos *et al*., this depression is expected to be the same for each Ln^3+^ ion used as scintillator centre in the same KGdP host^31^.

As described by Dorenbos *et al*.^32^, the polyphosphate compounds have a large fraction of strongly bonding phosphate atoms, which cause that the *D*(A) is amongst the smallest, and the crystal field splitting, ε_cfs_, and the bandgap amongst the largest of all oxide compounds. By comparing the values of the *D*(A) of Table 3, it can be observed how this value decreases as smaller is the alkaline ion (K > Na > Li); this tendency in the phosphates was already observed by Dorenbos^31^.

Table 3 shows the spectroscopic redshift, *D*(A), as well as the centroid shift, ε_c_, crystal field splitting, ε_cfs_, effective distance of Gd-O bond, R_eff_, and spectroscopic polarizability, α_sp_, for the type III KGdP host and others. *D*(A), ε_c_ and ε_cfs_ were calculated by using the optical absorption spectrum in Fig. 2; R_eff_ and α_sp_ were calculated considering the ε_c_, as in the reference^14^, and the crystallographic data of the undoped non-centrosymmetric KGdP^22^.

The crystal field splitting, ε_cfs_ (the energy difference between the 5*d*_1_ and the 5*d*_5_) for the non-centrosymmetric KGdP is smaller than in the centrosymmetric one. This fact does not follow the expected behaviour since ε_cfs_ is determined by the strength of the crystal field, related to the shape and the size of the coordination polyhedron, and considering the same polyhedral shape, the smaller the bond length and larger distortion, the higher the crystal field splitting^28^. Comparing the two hosts, the non-centrosymmetric KGdP crystal presents shorter bond length than the centrosymmetric one, and they have similar distortion in the cation site. This different crystal field suffered by the Ce^3+^ ions may be related to effects in the second coordination sphere.

The centroid shift, ε_c_ (energy difference between the centroid value of the 5*d* of the free electron and the one inside the crystal) may be related to the ligand polarization and has no dependence on the crystal field splitting value. As it can be seen in the same table, the ε_c_ for the non-centrosymmetric KGdP in comparison with the centrosymmetric one is higher. Taking into account the ligand polarization model^29^, the fact that the non-centrosymmetric KGdP has a ε_c_ value larger than the centrosymmetric one, should be attributed to the major contribution of the effective distances, R_eff_, in the value of centroid shift (ε_c_ ∝ R_eff_^−6^).

All discussed parameters until now are dependent of the crystalline host, so they are also related to the Ce^3+^ concentration in the crystal and temperature. The spectroscopic values for non-centrosymmetric KGdP have been calculated for KGd_0.996_Ce_0.004_(PO_3_)_4_ and at room temperature.

Moreover, as 4*f*→5*d* transitions are parity allowed transitions, it was also expected a high value of optical absorption cross section. The observed value is 370 × 10^−20^ cm^2^ at 302.5 nm for Ce^3+^ in the type III KGdP crystal. The 4*f*→4*f* transitions in lanthanide ions are very weakly influenced by the crystal field and for this reason the position of such absorption transitions of Gd^3+^ are the expected ones and have been labelled by using the Dieke’s diagram^10^.

#### Optical emission

**Figure 3:**
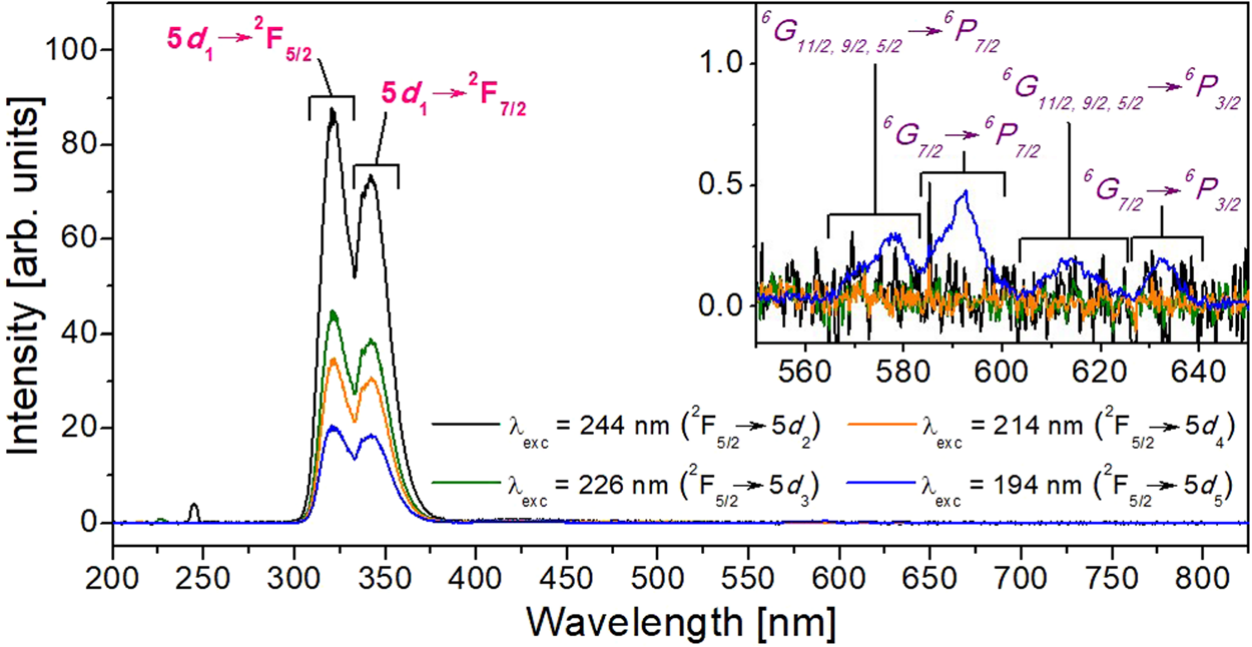
Optical emission spectra of KGd_0.996_Ce_0.004_(PO_3_)_4_ under 244, 226, 214 and 194 nm excitation. Labels in purple (inset) indicate the transitions of Gd^3+^ and labels in pink those of Ce^3+^.

Figure 3 shows the emission spectra of KCe_0.004_Gd_0.996_(PO_3_)_4_ under λ_exc_ = 244 nm (5.09 eV), 226 nm (5.49 eV), 214 nm (5.80 eV) and 194 nm (6.40 eV), which correspond to the excitation to the 5*d*_2_, 5*d*_3,_ 5*d*_4_ and 5*d*_5_ energy levels, respectively, of Ce^3+^ in the KGdP host. The intensities have been corrected by the excitation photon flux values. From this Figure, it can be seen an intense doublet peak centred at 322 and 342 nm corresponding to the 5*d*_1_→^2^F_5/2_ and *5d*_1_→^2^F_7/2_ transitions, respectively, of Ce^3+^ in KGdP. The energy difference between these two emission peaks is consistent with the energy difference between the 4*f* levels of Ce^3+^, ^2^F_5/2_ and ^2^F_7/2_ levels, as can be seen in the Dieke’s diagram^10^. This emission appears by exciting the sample under all these four different excitation wavelengths, in which the emission intensities are in accordance with the values of absorption coefficient for each excitation wavelength. The intensity of emission when the sample is excited at 194 nm is lower than when it is excited at 214 nm, despite their similar absorption coefficient values. This behaviour might be attributed to a depopulation of the 5*d*_5_ level of Ce^3+^ due to energy transfer to the 4*f* levels of Gd^3+^.

It is worth saying that the ^6^P_7/2_→^8^S_7/2_ transition of Gd^3+^ which should appear as a sharp emission around 311 nm, being common in many hosts^33–36^, is not present in the non-centrosymmetric KGd_1−x_Ce_x_(PO_3_)_4_ crystals, as observed in Fig. 3. The same behaviour is observed in the KGd_0.981_Ce_0.019_(PO_3_)_4_ and KGd_0.974_Ce_0.026_(PO_3_)_4_ crystals, referring to the emissions of Ce^3+^ and Gd^3+^ under the wavelength excitation 244, 226, 214 and 194 nm. By comparing the emission spectrum obtained under VUV excitation and that obtained under X-ray excitation of four highly similar hosts^2,14,37^ to our compound, it has been observed that the ^6^P_7/2_→^8^S_7/2_ transition of Gd^3+^ is not present under VUV excitation, and it does not appear by exciting the compound with X-rays, either. Thus, in type III Ce:KGdP, since the above mentioned 4*f*→4*f* transition of Gd^3+^ is not observed (Fig. 3), it is expected that it will not appear under X-ray excitation. This is a favourable feature for scintillator use because the emissions which are of interest in this application are based on 5*d*→4*f* transitions, instead of the 4*f*→4*f* transitions, since the 5*d* levels have very short decay times, in the order of nanoseconds. However, it should be noted that similar spectra are not always obtained when the compound is excited with VUV radiation and with X-rays.

From the work done by Zhong *et al*.^14^ similar results were observed in KGdP_4_O_12_ host with type B phase (space group: *C*2/*c*). The 5*d*_1_→^2^F_5/2_ and 5*d*_1_→^2^F_7/2_ transitions appear at 323 and 343 nm, respectively. When Ce:KGdP_4_O_12_ is excited at 193 nm (^2^F_5/2_→5*d*_5_), the emission of Gd^3+^ at 311 nm looks very weak as a hump in the band corresponding to the *5d*_1_→^2^F_5/2_ emission. Therefore, this emission of Gd^3+^ is extraordinarily rare in comparison with the emissions of Ce^3+^ in the type III KGdP. In a similar phosphates, this Gd^3+^ emission appears more intense than the emission peaks corresponding to *5d*_1_→^2^F_5/2,_^2^F_7/2_ transitions of Ce^3+^ in Ce:LiGdP_4_O_12_ (space group: *C*2/*c*) under 292 nm (^2^F_5/2_→5*d*_1_), 273 nm (^8^S_7/2_→^6^I_J_ Gd^3+^) and 188 nm (^2^F_5/2_→5*d*_5_) excitation, and in Ce:NaGdP_4_O_12_ (space group: *P*2_1_/*n*) under 298 nm (^2^F_5/2_→5*d*_1_) and 273 nm (^8^S_7/2_→^6^I_J_ Gd^3+^) excitation^14^.

**Figure 4:**
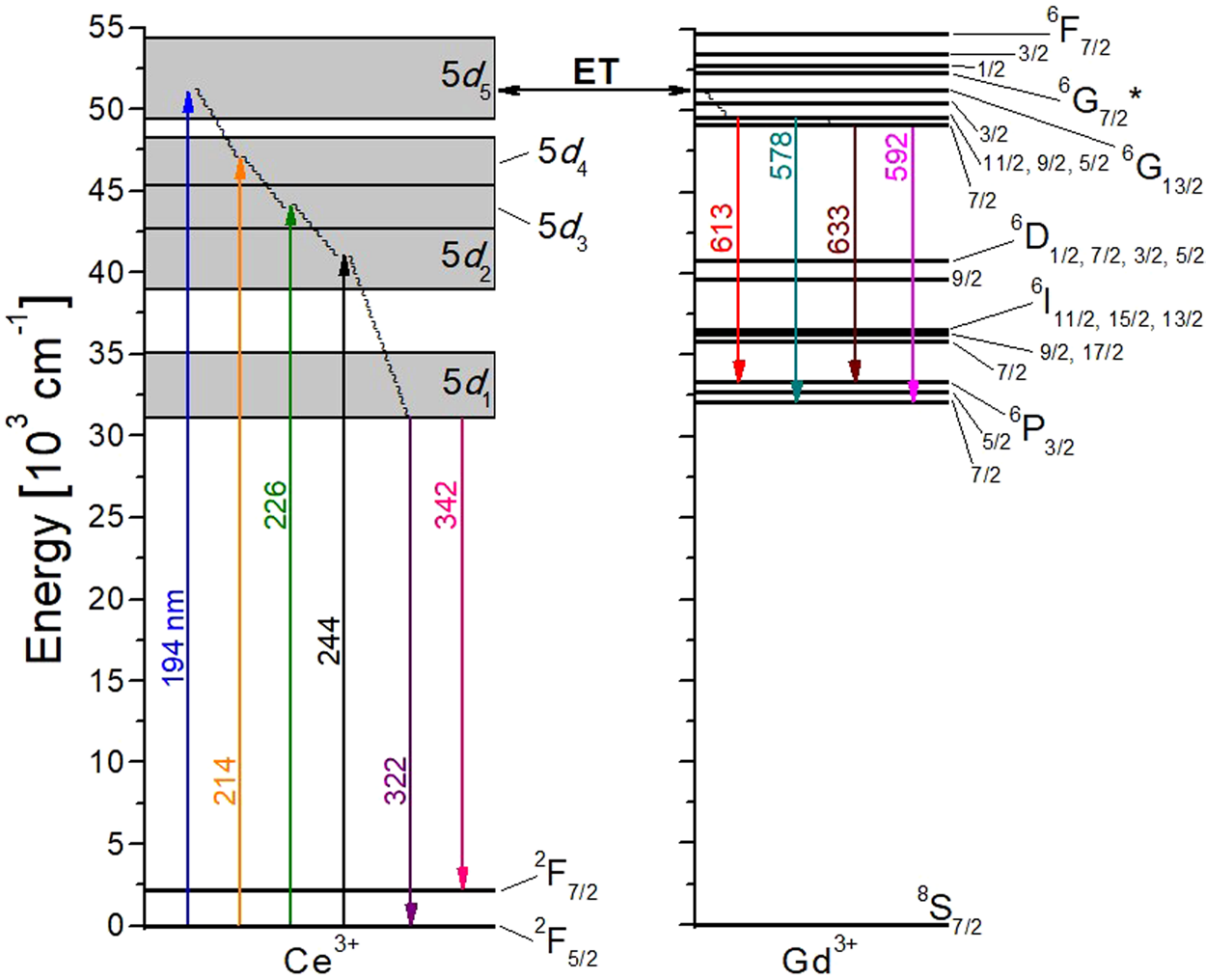
Energy levels diagram of Ce^3+^ and Gd^3+^ in KGd_0.996_Ce_0.004_(PO_3_)_4_ and the emission mechanism. ET = Energy Transfer.

In the inset of Fig. 3, it can be seen how several weak peaks appeared in the spectral range from 560 to 640 nm when the KCe_0.004_Gd_0.996_(PO_3_)_4_ sample is excited at 194 nm. These four weak peaks centred at 578, 592, 613 and 633 nm correspond to 4*f*→4*f* transitions of Gd^3+^, being the ^6^G_11/2,9/2,5/2_→^6^P_7/2_, ^6^G_7/2_→^6^P_7/2_, ^6^G_11/2,9/2,5/2_→^6^P_3/2_ and ^6^G_7/2_→^6^P_3/2_ transitions, respectively. This may occur since the ^8^S_7/2_→^6^G_13/2_ absorption transition of Gd^3+^ about 194 nm is overlapped with the ^2^F_5/2_→5*d*_5_ of Ce^3+^, as can be seen in Fig. 2. The energy levels of Gd^3+^ and Ce^3+^ in KGdP and the possible energy transfer mechanism are shown in Fig. 4. Returning to Figs 2 and 3, since the absorption from ^2^F_5/2_ ground state of Ce^3+^ to its 5*d*_1_ level appears at 302.5 nm and the emission corresponding to the 5*d*_1_→^2^F_5/2_ transition appears at 322 nm, the Stokes shift in the type III KGdP host is ΔS = 2002 cm^−1^. By observing the work done by Shalapska *et al*.^12^, the absorption peak corresponding to ^2^F_5/2_→5*d*_1_ transition of Ce^3+^ in Ce:NaPrP_4_O_12_ (space group: *P*2_1_/*n*) appear at 295 nm, and the 5*d*_1_→^2^F_5/2_ and 5*d*_1_→^2^F_7/2_ transitions appear as emissions at 310 and 328 nm. The characteristics of Ce^3+^-luminescence in LiYP_4_O_12_ host have also been studied by Shalapska *et al*.^38^. In this host, the absorption peaks corresponding to ^2^F_5/2_→5*d*_1_ transition of Ce^3+^ appear at 295 and the 5*d*_1_→^2^F_5/2_ and 5*d*_1_→^2^F_7/2_ transitions appear at 312 and 333 nm. From the experimental data mentioned, the Stokes shifts are ΔS(KGdP_4_O_12_) = 1614 cm^−1^, ΔS(NaPrP_4_O_12_) = 1640 cm^−1^ and ΔS(LiYP_4_O_12_) = 1847 cm^−1^. Thus, the Stokes shift of type III KGd(PO_3_)_4_ host is higher [ΔS = 2002 cm^−1^] than the calculated in the three hosts mentioned above, but still slightly below the most frequent Stokes shift measured in 240 different compounds, being 2200 cm^−1^ ^27^. The Stokes shift is induced by lattice relaxation at the excited states. Hence, the higher the value of Stokes shift is, the larger the relaxation of the electron at the excited state is before emitting electromagnetic radiation, resulting in higher non-radiative losses^39,40^. The Stokes shift is not dependent of the lanthanide ion, but only depends on the host. So, it can be extrapolated for future doping ions in the KGdP crystal^31^.

**Figure 5:**
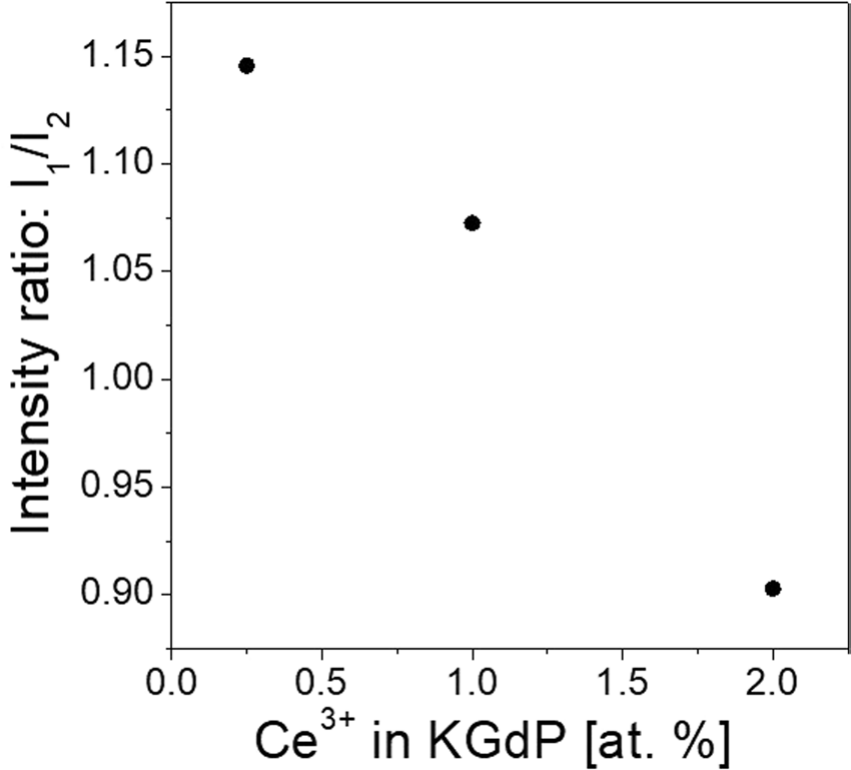
Intensity ratio of the emission peaks belonging to the 5*d*_1_ →^2^F_5/2_ transition (I_1_) versus the emission peaks belonging to 5*d*_1_→^2^F_7/2_ transition (I_2_) of Ce^3+^ under 226 nm excitation (^2^F_5/2_→5*d*_3_).

Moreover, it is also interesting to observe the intensity ratios of the peaks corresponding to 5*d*_1_→^2^F_5/2_ and 5*d*_1_→^2^F_7/2_ transitions of Ce^3+^, since it varies with the doping level. As the Ce^3+^ doping is higher, the peak corresponding to the transition from 5*d*_1_ to ^2^F_5/2_ becomes less intense compared to the transition to ^2^F_7/2_. The evolution of this ratio of intensities versus Ce^3+^ concentration can be seen in Fig. 5. This behaviour may be due to the presence of reabsorption of the emission corresponding to the *5d*_1_→^2^F_5/2_ transition by the neighbouring Ce^3+^ atoms.

**Figure 6:**
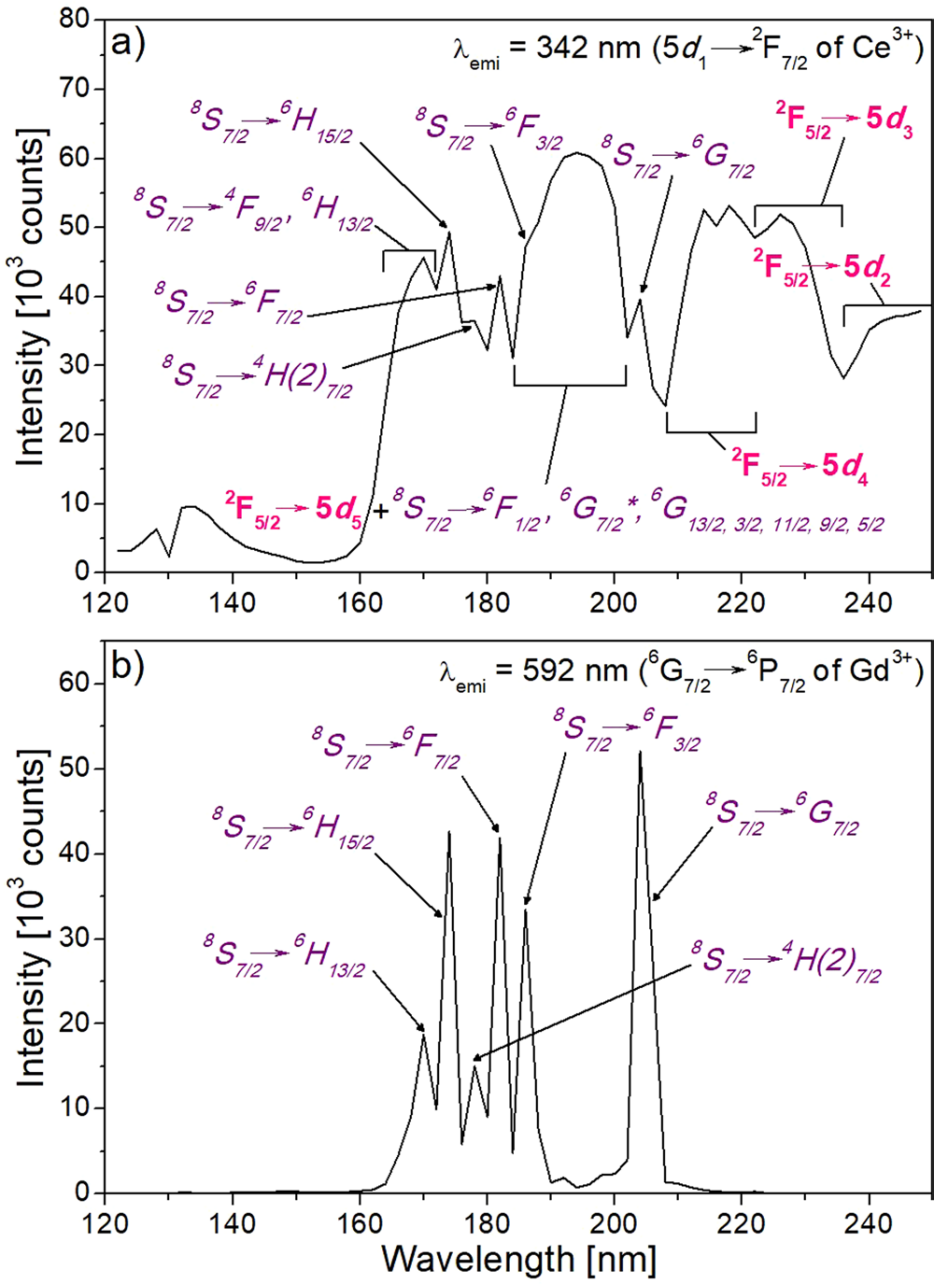
Excitation spectra of KCe_0.019_Gd_0.981_(PO_3_)_4_ for the emission wavelengths of (**a**) 342 nm (5*d*_1_→^2^F_7/2_ of Ce^3+^) and (**b**) 592 nm (^6^G_7/2_→^6^P_7/2_ of Gd^3+^). Labels in purple indicate the transitions of Gd^3+^ and labels in pink those of Ce^3+^.

Figure 6 shows the excitation spectrum of KGd_0.981_Ce_0.019_(PO_3_)_4_ at room temperature for the emission wavelengths λ_emi_ = 342 nm (5*d*_1_→^2^F_7/2_ of Ce^3+^) and λ_emi_ = 592 nm (^6^G_7/2_→^6^P_7/2_ of Gd^3+^) with the electronic transitions labelled. In the case of the excitation spectrum for the emission of Ce^3+^, it can be observed that these emissions take place when Ce^3+^ is excited and also when Gd^3+^ is excited. Therefore, this reaffirms that an energy transfer from Gd^3+^ to Ce^3+^ occurs. In addition, from the same spectrum (Fig. 6a), the complete band of the 5*d*_5_ level of Ce^3+^ has been observed complementing the assignment of 5*d* levels energy values in the absorption measurements.

From Fig. 6b, it can be seen how the emission at 592 nm is produced mainly when Gd^3+^ cations are directly excited. Similar behaviours have been observed for the emissions centred at 578, 613 and 633 nm, all corresponding to 4*f*→4*f* electronic Gd^3+^ transitions.

The energy of the exciton creation (*E*^ex^), that is, bound electron and hole pairs, in type III KGdP has been predicted that appears at 164 nm (7.57 eV) by the calculations explained below. First, the energy of the onset of the fundamental absorption (*E*^fa^) of type III KGdP has been established at 172 nm (7.22 eV) by overlapping a straight line along the ultraviolet absorption edge observed in the transmission spectrum of Nd:KGd(PO_3_)_4_ crystal^18^. This same procedure was used in the work done by Ueda *et al*.^41^. Then, the estimated value of *E*^ex^ has been obtained by adding 0.35 eV to *E*^fa^ since these measurements have been performed at room temperature^42^. Hence, the band corresponding to *E*^ex^ could appear centred at 164 nm in the excitation spectra (Fig. 6), although it would not be appreciated due to an overlapping with the ^8^S_7/2_→^4^F_9/2_ transition of Gd^3+^.

From the value of *E*^ex^, it can be calculated the approximate energy difference from the bottom of the conduction band to the top of the valence band (*E*_VC_); being approximately 1.08 times the energy of the exciton creation^42^. Therefore, the estimated value for type III KGdP is *E*_VC_ = 8.17 eV (152 nm).

#### Decay time measurements

**Figure 7:**
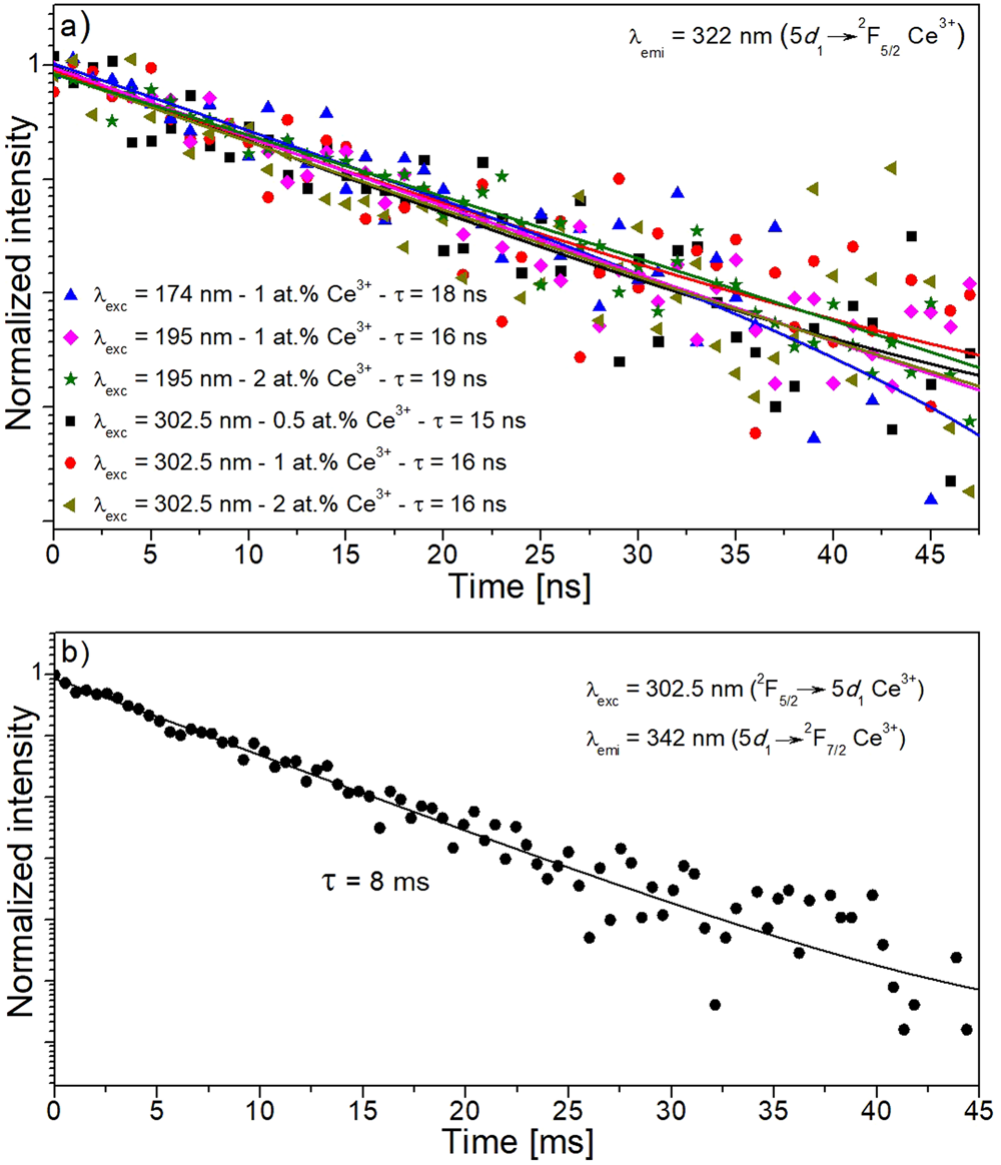
(**a**) Fast component of the luminescence decay curves of Ce^3+^-doped KGdP crystals with different doping concentrations and at different excitation wavelengths for λ_emi_ = 322 nm. (**b**) Slow component of the decay curve of KGd_0.974_Ce_0.026_(PO_3_)_4_ under excitation at 302.5 nm for λ_emi_ = 342 nm.

Ce^3+^-doped crystals were excited at 302.5 nm (^2^F_5/2_→5*d*_1_ of Ce^3+^), 194 nm (^8^S_7/2_→^6^G_13/2_ of Gd^3+^ and ^2^F_5/2_→5*d*_5_ of Ce^3+^) and 174 nm (^8^S_7/2_→^6^H_15/2_ of Gd^3+^). It should be noted that in order to gain in photon flux reaching to the sample, the excitation beam had a bandwidth of around 7%, being not purely monochromatic. Figure 7a shows the luminescence decay curves for the emission at λ_emi_ = 322 nm of Ce^3+^-doped KGdP crystals with different doping concentrations and at different excitation wavelengths. As can be seen in the Figure, the decay curves can be fitted by a single exponential decay. The lifetime obtained for 5*d*_1_ level of Ce^3+^ in this host is around 16 ns. No significant changes in the lifetime value were obtained either due to the change of the wavelength of excitation or the different Ce^3+^ concentration in the crystals.

This measured lifetime of the 5*d*_1_ level of Ce^3+^ in the KGdP crystal is similar or even shorter than the measured ones in other scintillator materials^3,12,14,43^. By comparing this value with the obtained in the centrosymmetric KGdP (space group: *C*2/*c*, 21.1 ns), the lifetime of the 5*d*_1_ level of Ce^3+^ in type III KGdP (space group: *P*2_1_) is significantly lower^14^.

It has been also observed that in type III Ce^3+^:KGdP crystals, the 5*d*_1_→^2^F_5/2_,^2^F_7/2_ decay is not only composed by a fast component, but also by a slow component. This fact was observed when the intensity of the emission does not reach zero value because of the short interpulse time in the synchrotron measurements. To measure in the appropriate magnitude this slow component, the lifetime was measured in a conventional fluorimeter. Figure 7b shows the slow component of this decay curve in the KGd_0.974_Ce_0.026_(PO_3_)_4_ crystal under excitation at λ_exc_ = 302.5 nm for the emission at λ_emi_ = 342 nm. In this Figure, it can be seen the exponential fit of the slow component resulting in a decay time around 8 ms. This slow component has been already observed in other Ce^3+^-doped phosphates, as in Ce:NaPrP_4_O_12_^12^, and Ce:CsGd(PO_3_)_4_^14^. In the first case, it was only observed at high energy excitations and it was attributed to trapping effect which makes the host-cerium energy transfer process more difficult, but in the case of Ce:CsGd(PO_3_)_4_, the slow component was observed when solely the Gd^3+^ ion was excited and not the Ce^3+^ ions. The long component in the Ce^3+^-doped non-centrosymmetric KGdP crystal has been observed when exciting at short wavelength values (Fig. 7a) and also when exciting directly to the 5*d*_1_ level (Fig. 7b). Therefore, the origin of this slow component could be attributed to some contribution of the trapping effect, but the contribution of the Gd^3+^ emission corresponding to the electronic transition ^6^P_7/2_→^8^S_7/2_ cannot be disregarded since the lifetime of this emitting ^6^P_7/2_ level of Gd^3+^ is of the same order (4.9 ms in NaY_0.80_Gd_0.20_PO_4_^34^ and 6.36 ms in NaGd(PO_3_)_4_^44^.

## Conclusions

Type III Ce^3+^-doped bulk single crystals have been grown by Top Seeded Solution Growth-Slow cooling technique from self-fluxes in high crystalline quality. Till a Ce^3+^ doping level of 2 at% substituting Gd^3+^ in solution, no effect of the cerium doping has been observed in the crystalline quality of the single crystals. The maximum optical absorption cross section has been measured for the electronic transition from the ground state of Ce^3+^ until the 5*d*_1_ level, being the value at 302.5 nm around 370 × 10^−20^ cm^2^. The spectroscopic redshift of the 5*d* energy levels of Ce^3+^ in the type III KGdP host is 16282 cm^−1^. Under ultraviolet excitation, a doublet emission peak centred at 322 and 342 nm has been observed in all grown crystals, corresponding to the 5*d*_1_→^2^F_7/2_ and 5*d*_1_→^2^F_5/2_ transitions of Ce^3+^, respectively. No important Gd^3+^ 4*f*→4*f* emissions have been clearly observed by exciting at this range. By last, the lifetime of the 5*d*_1_ level of Ce^3+^ in type III KGdP has been measured by setting the doublet emission peak and a lifetime around 16 ns has been obtained, which is similar or even shorter than the lifetime of the same level of Ce^3+^ in other hosts. In addition, the presence of a slow component has been observed by setting the same emission peak. The reported results confirm the potentiality of the Ce^3+^-doped type III KGd(PO_3_)_4_ crystals for scintillation applications.

## Experimental

### Single crystal growth

Ce^3+^:KGdP single crystals with non-centrosymmetric monoclinic structure (space group: *P*2_1_) have been grown from high temperature solutions. The solvent used was an excess of K_2_O and P_2_O_5_ (self-flux), so that it does not contain foreign ions that could be introduced as impurities in the crystalline structure. The compositions of the growth solutions, chosen on the basis of the primary crystallization region of KGdP in the K_2_O-Gd_2_O_3_-P_2_O_5_ ternary system, previously determined^22,45^, were K_2_O:((1−x)Gd_2_O_3_ + x Ce_2_O_3_):P_2_O_5_ = 36:4:60 (mol%) with x in the range 0–0.02. The solutions, with a weight of about 100 g, were prepared in a platinum cylindrical crucible of 40 mm in diameter. The reagents used were K_2_CO_3_ anhydrous (Alfa Aesar A. Johnson Matthey Company, 99%), NH_4_H_2_PO_4_ (Fluka Analytical, ≥99.0%) and both Gd_2_O_3_ and Ce_2_(CO_3_)_3_ from Aldrich with a purity of 99.9%.

Due to the relatively high level of dynamic viscosity of the solutions^25^, the mixing of the solution was produced by using high axial thermal gradient (3.0 K·mm^−1^ in depth), with the coolest point at the center of the solution surface (the solution density decreases with increasing the temperature), accompanied by the use of a platinum stirrer (20 mm in diameter) located at about 12 mm below the surface of the solution, rotating at 55 rpm. The growth was carried out on an undoped KGdP seed with a^*^ orientation with the *b* crystallographic direction tangential to the rotation direction, because in previous works it has been proved that this crystalline orientation gives excellent results in terms of crystal quality^16^. The seed, located in contact with the solution surface at about 10 mm from the rotation axis, was joined with the stirrer and rotated together^25^.

The saturation temperature of the solution was accurately determined by measuring the growth and dissolution rates of the KGdP seed as a function of the temperature. Beginning at the determined saturation temperature, cooling rates of 0.1 K·h^−1^ for the firsts 15 K and 0.05 K·h^−1^ for the next 10–15 K were applied to achieve the required supersaturation of the solution for crystal growth. After finishing the cooling ramps or when the crystal was big enough, it was slowly removed from the solution. To avoid thermal shocks in the crystal, it was cooled to room temperature inside the furnace at a rate of 20–25 K·h^−1^.

The composition of the crystals was determined by Electron Probe Microanalysis with Wavelength Dispersive Spectroscopy (EPMA-WDS) using a JEOL JXA-8230. The standard used to measure K, Gd, P and O was a KGdP single crystal, while the Ce content in the crystals was measured using CeO_2_ as standard. The measures were made with an accelerating voltage of 20 kV and a current of 20 nA. The measuring time for K, Gd, P and O was 10 s for peak and 5 s for background, while in the case of Ce the measuring time was 120 s for peak and 60 s for background, because of its low concentration in the crystals. K_α_ X-ray lines of K, P and O and L_α_ X-ray lines of Gd and Ce were used for the composition measurements. The dispersive crystals were PETJ for K, LIFH for Gd, PETL for P and Ce and LDE1 for O measurements. Under these conditions, the detection limit of Ce was 195 ppm.

### X-ray powder diffraction

The unit cell parameters of KGdP with Ce^3+^ doping up to 2 at.% in solution were determined by X-ray powder diffraction. The equipment used was a D5000 Siemens X-ray powder diffractometer in a θ-θ configuration with the Bragg-Brentano geometry. The measurements were carried out with step size of 0.03° and a step time of 7 s and recorded in the range 2θ = 10–70°. The unit cell parameters were refined using the TOPAS program^46^.

### Optical characterization

The grown crystals were cut in plates perpendicular to the crystallographic a*, *b* and c* directions with a diamond saw, lapped with Al_2_O_3_ suspension with particle size of 9 and 3 μm, successively, and polished with colloidal suspension of amorphous silicon dioxide with a mean particle size of 0.2 μm. These plates were used for optical absorption and emission studies. The optical absorption of Ce^3+^ in KGdP has been studied at room temperature using a 0.25 at.% Ce:KGdP plate parallel to the (001) crystallographic plane with a thickness of 0.11 mm and the equipment used was a CARY 5000 UV-Vis-NIR Spectrophotometer.

The emission spectroscopy of Ce^3+^-doped KGdP single crystals under vacuum ultraviolet excitation was carried out in the wavelength range from 120 to 248 nm (10–5 eV). Experiments were performed in the DESIRS beamline at SOLEIL Synchrotron (France, proposal num. 20151215, Standard). The samples were placed in a vacuum chamber which can be evacuated to around 10^−5^ bar. A Lithium Fluoride window at the entrance of the vacuum chamber separates it from the synchrotron line. The monochromatic synchrotron light reached perpendicularly to the KGdP plate. The emitted light from the sample was collected at 45° by a silica lens, focused at the entrance of an optical fibre connected to a Jaz spectrometer, Ocean Optics with a minimum spectral resolution of 0.3 nm. The emission spectra were recorded from 192 to 886 nm.

Lifetime measurements were also made in the DESIRS beamline at SOLEIL Synchrotron in a single bunch mode of operation to obtain pulsed radiation with full width at half-maximum pulse duration of 50 ps and interpulse time duration of 1.12 ms (proposal number 20161324, Standard). The crystals were placed at the same vacuum chamber, with the same configuration that in the emission measurements. The output emission of the crystal, focused with the same silica lens, was guided with an optical fibre to an ANDOR Shamrock 193i spectrograph (grating 150 lines/mm) coupled to an iStar Intensified Charge Coupled Device camera with fast response (DH734–18F-03 model). To increase the photon flux reaching the sample, once the vacuum in the chamber was enough high (at least 2 × 10^−5^ bars), the optical window between the vacuum chamber and the synchrotron line was removed. Long components of the time decays have been checked and measured by a conventional fluorimeter Cary Eclipse Fluorescence Spectrophotometer.
